# Circulating Tumor DNA-Based Detection of Microsatellite Instability and Response to Immunotherapy in Pancreatic Cancer

**DOI:** 10.3389/fphar.2020.00023

**Published:** 2020-02-10

**Authors:** Saivaishnavi Kamatham, Faisal Shahjehan, Pashtoon Murtaza Kasi

**Affiliations:** ^1^Department of Cancer Biology/Pathology, Wayne State University, Detroit, MI, United States; ^2^Department of Internal Medicine, Conemaugh Memorial Medical Center, Johnstown, PA, United States; ^3^Department of Hematology/Oncology, University of Iowa, Iowa City, IA, United States

**Keywords:** circulating tumor DNA, microsatellite instability, pancreatic cancer, immunotherapy, pembrolizumab

## Abstract

Pancreatic cancer is an aggressive malignancy with poor survival. Research has indicated the association of few genetic aberrations with pancreatic cancer. The data regarding the prevalence of microsatellite instability in pancreatic cancer is diverse and controversial. However, it could be an actionable target in pancreatic cancer especially due to availability of immune checkpoint inhibitors which has demonstrated promising results in different types of cancers. We present a case of pancreatic cancer whose microsatellite instability status was identified on liquid biopsy (circulating tumor DNA testing). Our patient showed a dramatic ongoing durable response to immunotherapy. We were able to do serial monitoring with liquid biopsy that showed clinical utility and validity.

## Background

Pancreatic cancer is a challenging disease with unfavorable outcomes. Pancreatic ductal adenocarcinoma (PDAC) constitutes around 90% of all malignant pancreatic cancers (Hackeng et al., 2016). As opposed to other common cancers, there has been a rise in incidence and mortality rates of PDAC (Ryerson et al., 2016). According to the American Cancer Society, it is the fourth leading cause of cancer-related mortality in both males and females with an estimated number of new cases and deaths in the United States in 2019 as 56,770 and 45,750, respectively (Siegel et al., 2019). PDAC is associated with poor prognosis, having a 5-year survival rate of 8%, owing to its presentation as an advanced disease, being resistant to different drug regimens and a distinct tumor microenvironment with condensed desmoplasia (Mahadevan and Von Hoff, 2007; Yachida and Iacobuzio-Donahue, 2009; Erkan et al., 2010; Erkan et al., 2012; Liu et al., 2017). Chemotherapy, both in adjuvant and neoadjuvant settings, has remained the treatment of choice for most patients with PDAC. However, it has not been helpful in a significant improvement of survival of these patients. This leaves surgery as the only curative option but fewer than 20% of patients present with a resectable disease at diagnosis (DeWitt et al., 2004). The unavailability of a useful diagnostic and prognostic biomarker has always been a concern for PDAC (Herreros-Villanueva and Bujanda, 2016).

PDAC could be associated with several targets including *KRAS*, *TP53*, *TGF-β*, *WNT*, *NOTCH*, *SMAD4*, *CDKN2A*, *ARID1A*, *MLL3*, and *TGFBR2* (Bailey et al., 2016; Chou et al., 2018). Important are aberrations in BRCA and DNA repair for whom PARP-inhibitors are now an option. Small proportion (1%–2%) of PDAC is also associated with microsatellite instability (MSI) (Barrett et al., 2017; Humphris et al., 2017; Hu et al., 2018). MSI results from mismatch repair deficiency (dMMR) and consists of repetitive 1–6 base pairs of DNA (Umar and Kunkel, 1996; Humphris et al., 2017). dMMR or loss of functional ability of any of the mismatch repair proteins (MLH1, MSH2, MSH6, and PMS2) hinders the effective DNA replication process. Research has indicated that cancers with dMMR and MSI-H respond very well to immune checkpoint inhibitors. This implies that targeting the immune checkpoints including programmed cell death-1 (PD-1) and cytotoxic T lymphocyte antigen-4 (CTLA-4) encourages T cells to fight cancer cells. This has been well-elaborated for melanoma and lung cancer, but data is lacking for PDAC.

Herein, we report a case of PDAC whose MSI-H status was identified on liquid biopsy which is also known as circulating tumor DNA (ctDNA) testing. Our patient showed a dramatic response to immunotherapy which was again assessed on ctDNA assays.

## Case Presentation

Patient is an 81-year old female who had unrelenting pain in her stomach in January 2018. She had an ultrasound done by her gastroenterologist that showed a mass in the pancreatic head versus body. A follow-up CT scan confirmed the ultrasound findings and also showed one possible enlarged lymph node. This was followed up by MRI at Mayo Clinic, Florida in March 2018, which showed locally advanced pancreatic mass centered at the junction of the body and tail measuring 5.1 × 6.4 × 5.2 cm. The results also showed encasement of the first jejunal arterial and venous branches with adjacent mass effect causing narrowing of the superior mesenteric vein confluence. Enlarged right common iliac artery chain lymph node measuring 1.4 × 1.1 cm and indeterminate lesions in the posterior aspect of the vertebral body at T9 were noted.

A baseline ctDNA test was obtained, which is at present part of our standard of care at Mayo Clinic, Florida for patients with GI malignancies. Testing is performed through commercially available platforms. In this particular patient, this was done through Guardant360 that showed *SMAD4 R361H*, *TP53R213L*, *KRAS G12D*, *RET A640A*, *KIT K412K*, *NTRK3 R630G*, *ARID1A G1711fs* and the highest variant allele fraction was noted to be 2.2% as shown in **Table 1**. She was started on neoadjuvant chemotherapy with gemcitabine and paclitaxel since surgery was not an option, but there was not much response in her repeat scans in May 2018, though there was a decrease in the highest variant allele fraction to 1.2%. She was then switched to 5-fluorouracil with nanoliposomal irinotecan (5-FU was held due to *DPD* heterozygosity on pharmacogenomics testing). Repeat CT scan in July 2018 showed decrease in the size of a mass in the pancreas which measures 3.4 × 3.1 cm compared with 6.5 × 5.1 cm. Moreover, highest variant allele fraction dropped down to 0.8% on ctDNA testing in July 2018. Chemoradiation with capecitabine was added to her treatment plan in August 2018.

**Table 1:** Serial circulating tumor DNA evaluation in our patient with MSI-H/dMMR pancreatic ductal adenocarcinoma and excellent response to pembrolizumab. As noted, the circulating tumor DNA in the *SMAD4 R361H* mutation has gone down from 2.2% to 0%.

Serial ctDNA(liquid biopsy)testing results						
	March 2018	May 2018	July 2018	Dec 2018	Jan 2019	Feb 2019
Highest Variant Allele Fraction						
	2.2%	1.2%	0.8%	0.7%	0.5%	0.4%
***Clonal Mutations***						
*SMAD4 R361H*	2.2%	0.9%	0.6%	0.7%	0%	0%
*TP53 R213L*	1.9%	0.8%	0.5%	0.6%	0%	0%
*KRAS G12D*	1.9%	1.2%	0.4%	0.4%	0%	0%
*RET A640A*	1.6%	0.5%	0.5%	0.5%	0%	0%
*KIT K412K*	1.5%	0.4%*	0.3%*	0.6%	0%	0%
*NTRK3 R630G*	1.4%	0.6%	0.4%	0.4%	0%	0%
*ARID1A G1711fs*	1.1%	0.8%	0.3%*	0.3%*	0%	0%
***Subclonal Mutations***						
*MTOR I486V*	1.0%	0.1%	0.3%	0.4%*	0%	0%
*BRCA2 A1572T*	0.9%	1.2%*	0.8%*	0.5%*	0.5%	0.4%
*HNF1A A209T*	0.9%	0.4%	0.4%	0.3%	0%	0%
*ARID1A G1847G*	0.9%	0%	0%	0%	0%	0%
*DDR2 K699*	0.7%	0%	0.2%	0%	0%	0%
*MPL S505N*	0.5%	0%	0.2%	0%	0%	0%
*PIK3CA W11R*	0.4%	0%	0%	0.3%	0%	0%
*MET N1081S*	0.2%	0%	0%	0.2%	0%	0%
*ATM G2675*	0.2%	0%	0%	0%	0%	0%
*EGFR E543G*	0.1%	0%	0%	0%	0%	0%

***MSI status***	ND	ND	ND	**high**	ND	ND
ǂ ***Concordance with tissue biopsy –* clinical concordance: yes clonal concordance: yes MSI-high detection: yes**						

***Treatment***	**Neoadjuvant chemotherapy (gemcitabine and paclitaxel/5-fluorouracil with nanoliposomal irinotecan)**			**Immunotherapy (Pembrolizumab)**		
***Tumor markers***						
*CEA (ng/ml)*	15	11.4	16.2	22.1	8.7	3.3
*CA-19-9 (Units/ml)*	5	4	6	6	7	6

50% of the highest variant allele fraction value has been used to differentiate clonal from sub clonal mutations. *indicates change from clonal to Subclonal and vice-versa; ND, Not Detected.

ǂBiomarker profiling on tissue sample detected SMAD4 R361H, KRAS G12D, ARID1A G1711fs mutations and MSI-high status. Therefore, ctDNA (liquid biopsy testing) is concordant with tissue biopsy testing in this patient.

**Figure 1:**
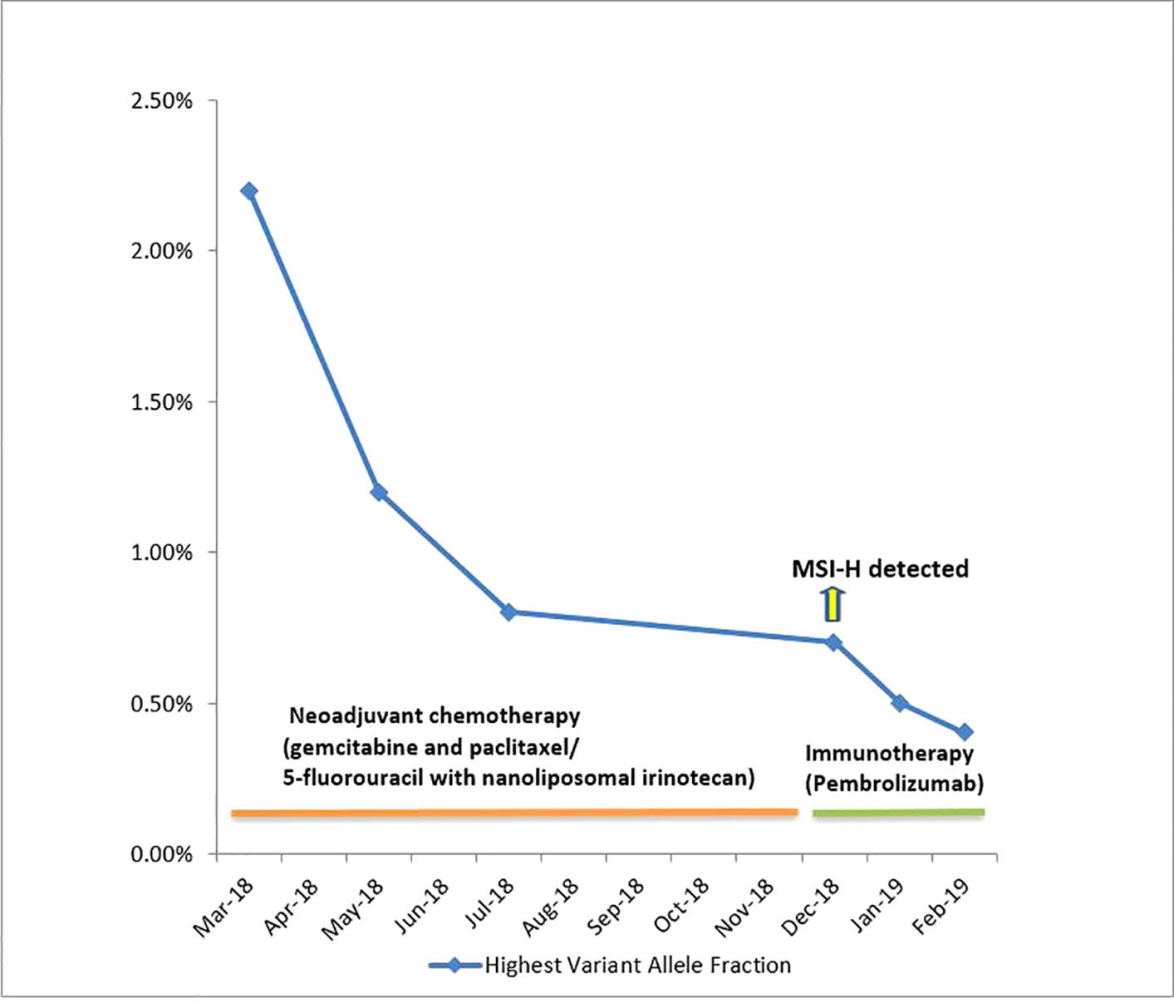
Serial liquid biopsies and changes in Highest Variant Allele Fractions of ctDNA over the course of therapy. MSI-H detected in ctDNA testing done in December 2018. MSI-H was not detected in ctDNA reports of January 2019 and February 2019.

In December 2018, CT scan of the chest showed a new right lower lung lobe nodule suspicious for metastatic disease along with a persistent mass in the body of the pancreas. In the repeat ctDNA testing in December 2018, she was noted to be MSI-High/mismatch repair deficient (dMMR). Furthermore, mismatch repair immunohistochemistry on the tissue sample showed loss of MLH1 and PSM2 proteins. Therefore, she was considered a great candidate for immunotherapy pembrolizumab on-label and was started on it. Dramatic improvement was noticed within 4 weeks of treatment with pembrolizumab and repeat ctDNA testing in January 2019 showed loss of all the above noted mutations. Furthermore, the highest variant allele fraction dropped down to 0.5% and patient continues to be on this therapy as depicted in **Figure 1**.

## Discussion

The management of PDAC is a complex task. To begin with, tissue biopsy has been the diagnostic test of choice historically which is a cumbersome technique. Moreover, it is troublesome for the patients to have repeated serial tissue biopsies in order to monitor the response to therapy. In the past decade, liquid biopsy has emerged as a noninvasive and patient-convenient technique that has demonstrated the clinical utility and validity for various cancers. However, it has not been widely incorporated into clinical practice for the management of all types of cancers especially PDAC because of sparsity of evidence. Our case showed the promising role of liquid biopsy not only in detection of the underlying genetic aberration but also in determination of the treatment response in PDAC.

The available data on the prevalence of dMMR and MSI-H in PDAC is limited and heterogeneous. Previous studies have demonstrated the existence of MSI-H in PDAC, however, the findings were controversial. Yamamoto et al. investigated 103 PDAC patients and the reported incidence of MSI-H in their cohort was 16%. The researchers also reported the finding of MSI-H in 100% of their patients with hereditary PDAC (n = 3) (Yamamoto et al., 2001). Goggins et al. studied 82 xenografts of pancreatic carcinoma and reported that 3.7% of their specimens had MSI-H (Goggins et al., 1998). Ouyang et al. investigated 60 pancreatic cancer patients and found MSI-H in 9 (15%) patients of their cohort (Ouyang et al., 1998). Venkatasubbarao et al. reported the presence of MSI-H in 4 (29%) out of their 14 surgically resected samples of pancreatic adenocarcinoma (Venkatasubbarao et al., 1998). Recently, Hu et al. investigated 833 PDAC patients and found dMMR in 7 (0.8%) patients of their cohort (Hu et al., 2018). The summary of studies looking at pancreatic carcinoma and MSI-H/dMMR expression are demonstrated in **Table 2**. The disagreement in prevalence of MSI-H in PDAC might be due to confounding factors including differences in sample sizes, test techniques and diversity of tumor histology (Macherla et al., 2018).

**Table 2:** Summary of studies looking at pancreatic carcinoma and MSI-H/dMMR expression.

Study	Year	Study group	MSI/MMR status	Data source
Singhi et al. (2019)	2019	3,594 PDAC samples	MSI-H was detected in 0.5% of samples	International cohort
Hu et al. (2018)	2018	833 pancreatic adenocarcinoma patients	7 (0.8%) patients had dMMR, all 7 dMMR patients had lynch syndrome	Multiple hospitals, USA
Lupinacci et al. (2018)	2018	445 pancreatic ductal adenocarcinoma tissue samples	1.6% samples were dMMR	Multiple centers
Humphris et al. (2017)	2017	385 pancreatic cancer tissue samples	1% specimens were dMMR	Multiple centers of Australia, Germany, UK and USA
Connor et al. (2017)	2017	160 pancreatic adenocarcinoma cases from 154 patients (148 primary, 12 metastases)	4 cases were dMMR (3 had germline and 1 had somatic mutations in MMR genes)	International Cancer Genome Consortium data portal
Grant et al. (2015)	2015	290 pancreatic ductal adenocarcinoma patients	4 (1.38%) patients were dMMR	Ontario Pancreas Cancer Study (Ontario population-based registry)
Riazy et al. (2015)	2015	265 pancreatic ductal adenocarcinoma cases	41 (15%) were dMMR	Vancouver Coastal Health Region
Mitsuhashi et al. (2015)	2015	282 pancreatic ductal adenocarcinoma patients	None of the patients had MSI-H	3 hospitals database, Japan
Liu et al. (2014)	2014	36 acinar cell carcinoma of pancreas cases	5 (14%) patients were dMMR	Single academic center, USA
Ottenhof et al. (2012)	2012	78 pancreatic adenocarcinoma patients	13% tumors were dMMR	3 cancer treatment centers, Netherlands
Maple et al. (2005))	2005	35 pancreatic cancer patients	3 (8.6%) were dMMR who also had lynch syndrome	Single academic center, USA
Nakata et al. (2003)	2003	55 pancreatic carcinoma patients	4 (7.3%) tumors had abnormal hMS2-negative staining	Single university medical center, Japan
Yamamoto et al. (2001)	2001	103 pancreatic ductal adenocarcinoma (100 sporadic, 3 hereditary) patients	13% patients had MSI-H among sporadic PDAC group; 100% of hereditary PDAC patients had MSI-H	Single university medical center, Japan
Goggins et al. (1998)	1998	82 xenografted pancreatic carcinomas	3 (3.7%) specimens had MSI-H	Single academic center, USA
Ouyang et al. (1998)	1998	60 pancreatic cancer patients	9 (15%) patients had MSI-H	Multiple hospitals, Japan
Venkatasubbarao et al. (1998)	1998	14 surgically resected pancreatic adenocarcinoma tissue samples	4 (29%) had MSI-H	Single university medical center, USA

Our patient demonstrated a phenomenal response to immunotherapy. The tumor mutation burden pre- and post-treatment was well-picked on ctDNA assays. Recent research has highlighted the clinical utility and validity of ctDNA testing in the management of various gastrointestinal cancers including PDAC (Shahjehan et al., 2019). Our findings also suggest that serial monitoring of tumor mutation burden can serve as a potential prognostic biomarker in pancreatic cancer. Previously, this was not feasible before the implication of ctDNA testing. The advent of immune checkpoint inhibitors has revolutionized the management of various MSI-H/dMMR tumors especially non-small cell lung carcinoma and melanoma. In May 2017, the United States Food and Drug Administration (FDA) approved pembrolizumab, a PD-1 inhibitor, for the treatment of MSI-H/dMMR solid tumors regardless of site of origin or histology that didn’t respond or have metastasized after the introduction of first line agents (Lemery et al., 2017). The role of immunotherapy in PDAC has been studied in various clinical trials, however, no objective/complete response was achieved (Brahmer et al., 2012; Patnaik et al., 2015). The potential factors responsible for resistance of PDAC to immunotherapy could be low immunogenicity, decreased tumor mutation burden and inherent quality of being unlikely to be detected by the immune system (Evans et al., 2016).

There are several ongoing clinical trials that are investigating the role of various anti-PD-1/PD-L1 agents including pembrolizumab, nivolumab, etc. as monotherapy as well as combination regimens in PDAC. A recent phase I study investigated the role of a combination regimen consisting of a tumor-associated macrophage-targeting agent cabiralizumab and the anti-PD-1 nivolumab in metastatic PDAC. The combination regimen demonstrated a confirmed objective response in 4 of 31 (13%) patients. The researchers also revealed that all of these 4 patients with confirmed objective response had MSI-H and didn’t show response to anti‒PD-1 or PD-L1 monotherapy (NCT02526017) (Wainberg ZA et al., 2017).

Le et al. investigated the efficacy of PD-1 blockade in the management of 12 types of solid tumors including pancreatic cancer in a multicenter phase 2 study. They enrolled all dMMR cancer patients (n = 86) who had at least one prior therapy and developed a progressive disease. The estimated objective and complete response rates were 53% and 21%, respectively. Of note, the subset analysis for pancreatic cancer (n = 8) demonstrated an objective response rate of 62%. The researchers indicated that the complete and partial responses were attained in 2 (25%) and 3 (37%) pancreatic cancer patients respectively. For the colorectal cancer (n = 40), their results showed the objective and complete response rates of 52% and 12%, respectively (NCT01876511). The study is still ongoing and manifests the value of immune checkpoint inhibitors in pancreatic cancer (Le et al., 2017).

## Conclusion and Future Directions

Pancreatic cancer is a lethal cancer with poor outcomes in spite of the recent breakthroughs in combination chemotherapy regimens. Immune checkpoint inhibitors have exhibited strong responses in several MSI-H solid tumors but there is lack of evidence regarding pancreatic cancer. Liquid biopsy could be incorporated in the management of these patients to record serial assessments of tumor mutation burden and to detect microsatellite instability, where obtaining tissue is often very difficult.

## Data Availability Statement

All datasets generated for this study are included in the article/supplementary material.

## Ethics Statement

Written informed consent was obtained from the patient for the publication of this case report.

## Author Contributions

SK and FS drafted the initial draft manuscript with guidance from PK. Author PK revised the manuscript and further edited by all the authors. All authors approved the final draft for publication.

## Conflict of Interest

The authors declare that the research was conducted in the absence of any commercial or financial relationships that could be construed as a potential conflict of interest.

## References

[B1] Bailey P. Chang D. K. Nones K. Johns A. L. Patch A.-M. Gingras M.-C (2016). Genomic analyses identify molecular subtypes of pancreatic cancer. Nature 531 (7592), 47–52. 10.1038/nature16965 26909576

[B2] Barrett M. T. Deiotte R. Lenkiewicz E. Malasi S. Holley T. Evers L (2017). Clinical study of genomic drivers in pancreatic ductal adenocarcinoma. Br. J. Cancer 117 (4), 572–582. 10.1038/bjc.2017.209 28720843 PMC5558689

[B3] Brahmer J. R. Tykodi S. S. Chow L. Q. Hwu W. J. Topalian S. L. Hwu P (2012). Safety and activity of anti-PD-L1 antibody in patients with advanced cancer. N. Engl. J. Med. 366 (26), 2455–2465. 10.1056/NEJMoa1200694 22658128 PMC3563263

[B4] Chou A. Froio D. Nagrial A. M. Parkin A. Murphy K. J. Chin V. T (2018). Tailored first-line and second-line CDK4-targeting treatment combinations in mouse models of pancreatic cancer. Gut 67 (12), 2142–2155. 10.1136/gutjnl-2017-315144 29080858 PMC6241608

[B5] Connor A. A. Denroche R. E. Jang G. H. Timms L. Kalimuthu S. N. Selander I (2017). Association of distinct mutational signatures with correlates of increased immune activity in pancreatic ductal adenocarcinoma. JAMA Oncol. 3 (6), 774–783. 10.1001/jamaoncol.2016.3916 27768182 PMC5824324

[B6] DeWitt J. Devereaux B. Chriswell M. McGreevy K. Howard T. Imperiale T. F (2004). Comparison of endoscopic ultrasonography and multidetector computed tomography for detecting and staging pancreatic cancer. Ann. Intern. Med. 141 (10), 753–763. 10.7326/0003-4819-141-10-200411160-00006 15545675

[B7] Erkan M. Reiser-Erkan C. Michalski C. W. Kleeff J (2010). Tumor microenvironment and progression of pancreatic cancer. Exp. Oncol. 32 (3), 128–131. 21403605

[B8] Erkan M. Reiser-Erkan C. Michalski C. W. Kong B. Esposito I. Friess H. (2012). The impact of the activated stroma on pancreatic ductal adenocarcinoma biology and therapy resistance. Curr. Mol. Med. 12 (3), 288–303. 10.2174/156652412799218921 22272725

[B9] Evans M. S. Diamond M. S. Rech A. J. Chao T. Richardson M. W. Lin J. H (2016). Lack of immunoediting in murine pancreatic cancer reversed with neoantigen. JCI Insight 1 (14). 10.1172/jci.insight.88328 PMC5026128 27642636

[B10] Goggins M. Offerhaus G. J. Hilgers W. Griffin C. A. Shekher M. Tang D. (1998). Pancreatic adenocarcinomas with DNA replication errors (RER+) are associated with wild-type K-ras and characteristic histopathology. poor differentiation, a syncytial growth pattern, and pushing borders suggest RER+. Am. J. Pathol. 152 (6), 1501–1507. 9626054 PMC1858440

[B11] Grant R. C. Selander I. Connor A. A. Selvarajah S. Borgida A. Briollais L (2015). Prevalence of germline mutations in cancer predisposition genes in patients with pancreatic cancer. Gastroenterology 148 (3), 556–564. 10.1053/j.gastro.2014.11.042 25479140 PMC4339623

[B12] Hackeng W. M. Hruban R. H. Offerhaus G. J. Brosens L. A. (2016). Surgical and molecular pathology of pancreatic neoplasms. Diagn. Pathol. 11 (1), 47. 10.1186/s13000-016-0497-z 27267993 PMC4897815

[B13] Herreros-Villanueva M. Bujanda L. (2016). Non-invasive biomarkers in pancreatic cancer diagnosis: what we need versus what we have. Ann. Transl. Med. 4 (7), 134. 10.21037/atm.2016.03.44 27162784 PMC4842402

[B14] Hu Z. I. Shia J. Stadler Z. K. Varghese A. M. Capanu M. Salo-Mullen E (2018). Evaluating mismatch repair deficiency in pancreatic adenocarcinoma: challenges and recommendations. Clin. Cancer Res. 24 (6), 1326–1336. 10.1158/1078-0432.CCR-17-3099 29367431 PMC5856632

[B15] Humphris J. L. Patch A. M. Nones K. Bailey P. J. Johns A. L. McKay S (2017). Hypermutation in pancreatic cancer. Gastroenterology 152 (1), 68–74.e2. 10.1053/j.gastro.2016.09.060 27856273

[B16] Le D. T. Durham J. N. Smith K. N. Wang H. Bartlett B. R. Aulakh (2017). Mismatch repair deficiency predicts response of solid tumors to PD-1 blockade. Science 357 (6349), 409–413. 10.1126/science.aan6733 28596308 PMC5576142

[B17] Lemery S. Keegan P. Pazdur R. (2017). First FDA approval agnostic of cancer site - when a biomarker defines the indication. N. Engl. J. Med. 377 (15), 1409–1412. 10.1056/NEJMp1709968 29020592

[B18] Liu W. Shia J. Gönen M. Lowery M. A. O'Reilly E. M. Klimstra D. S (2014). DNA mismatch repair abnormalities in acinar cell carcinoma of the pancreas: frequency and clinical significance. Pancreas 43 (8), 1264–1270. 10.1097/MPA.0000000000000190 25058881

[B19] Liu Q. Liao Q. Zhao Y. (2017). Chemotherapy and tumor microenvironment of pancreatic cancer. Cancer Cell Int. 17, p. 68. 10.1186/s12935-017-0437-3 PMC5498917 28694739

[B20] Lupinacci R. M. Goloudina A. Buhard O. Bachet J. B. Maréchal R. Demetter P (2018). Prevalence of microsatellite instability in intraductal papillary mucinous neoplasms of the pancreas. Gastroenterology 154 (4), 1061–1065. 10.1053/j.gastro.2017.11.009 29158190

[B21] Macherla S. Laks S. Naqash A. R. Bulumulle A. Zervos E. Muzaffar M. (2018). Emerging Role of Immune Checkpoint Blockade in Pancreatic Cancer. Int. J. Mol. Sci. 19 (11). 10.3390/ijms19113505 PMC6274962 30405053

[B22] Mahadevan D. Von Hoff D. D. (2007). Tumor-stroma interactions in pancreatic ductal adenocarcinoma. Mol. Cancer Ther. 6 (4), 1186–1197. 10.1158/1535-7163.MCT-06-0686 17406031

[B23] Maple J. T. Smyrk T. C. Boardman L. A. Johnson R. A. Thibodeau S. N. Chari S. T (2005). Defective DNA mismatch repair in long-term (> or = 3 years) survivors with pancreatic cancer. Pancreatology 5 (2-3), 220–7; discussion 227-8. 10.1159/000085275 15855819

[B24] Mitsuhashi K. Nosho K. Sukawa Y. Matsunaga Y. Ito M. Kurihara H (2015). Association of Fusobacterium species in pancreatic cancer tissues with molecular features and prognosis. Oncotarget 6 (9), 7209–7220. 10.18632/oncotarget.3109 25797243 PMC4466679

[B25] Nakata B. Wang Y. Q. Yashiro M. Ohira M. Ishikawa T. Nishino H. (2003). Negative hMSH2 protein expression in pancreatic carcinoma may predict a better prognosis of patients. Oncol. Rep. 10 (4), 997–1000. 10.3892/or.10.4.997 12792759

[B26] Ottenhof N. A. Morsink F. H. Ten Kate F. van Noorden C. J. Offerhaus G. J (2012). Multivariate analysis of immunohistochemical evaluation of protein expression in pancreatic ductal adenocarcinoma reveals prognostic significance for persistent Smad4 expression only. Cell Oncol. (Dordr) 35 (2), 119–126. 10.1007/s13402-012-0072-x 22351431 PMC3306569

[B27] Ouyang H. Furukawa T. Abe T. Kato Y. Horii A (1998). The BAX gene, the promoter of apoptosis, is mutated in genetically unstable cancers of the colorectum, stomach, and endometrium. Clin. Cancer Res. 4 (4), 1071–1074. 9563904

[B28] Patnaik A. Kang S. P. Rasco D. Papadopoulos K. P. Elassaiss-Schaap J. Beeram M (2015). Phase I study of pembrolizumab (MK-3475; Anti-PD-1 monoclonal antibody) in patients with advanced solid tumors. Clin. Cancer Res. 21 (19), 4286–4293. 10.1158/1078-0432.CCR-14-2607 25977344

[B29] Riazy M. Kalloger S. E. Sheffield B. S. Peixoto R. D. Li-Chang H. H. Scudamore C. H (2015). Mismatch repair status may predict response to adjuvant chemotherapy in resectable pancreatic ductal adenocarcinoma. Mod. Pathol. 28 (10), 1383–1389. 10.1038/modpathol.2015.89 26226846

[B30] Ryerson A. B. Eheman C. R. Altekruse S. F. Ward J. W. Jemal A. Sherman R. L (2016). Annual report to the nation on the status of cancer, 1975-2012, featuring the increasing incidence of liver cancer. Cancer 122 (9), 1312–1337. 10.1002/cncr.29936 26959385 PMC4840031

[B31] Shahjehan F. Kamatham S. Kasi P. M. (2019). Role of circulating tumor dna in gastrointestinal cancers: update from abstracts and sessions at ASCO 2018. Front. Oncol. 9, 358–358. 10.3389/fonc.2019.00358 31139561 PMC6519295

[B32] Siegel R. L. Miller K. D. Jemal A. (2019). Cancer statistics, 2019. CA Cancer J. Clin. 69 (1), 7–34. 10.3322/caac.21551 30620402

[B33] Singhi A. D. George B. Greenbowe J. R. Chung J. Suh J. Maitra A (2019). Real-time targeted genome profile analysis of pancreatic ductal adenocarcinomas identifies genetic alterations that might be targeted with existing drugs or used as biomarkers. Gastroenterology 156 (8), 2242–2253.e4. 10.1053/j.gastro.2019.02.037 30836094

[B34] Umar A. Kunkel T. A. (1996). DNA-replication fidelity, mismatch repair and genome instability in cancer cells. Eur. J. Biochem. 238 (2), 297–307. 10.1111/j.1432-1033.1996.0297z.x 8681938

[B35] Venkatasubbarao K. Ahmed M. M. Swiderski C. Harp C. Lee E. Y. McGrath P. (1998). Novel mutations in the polyadenine tract of the transforming growth factor beta type II receptor gene are found in a subpopulation of human pancreatic adenocarcinomas. Genes Chromosomes Cancer 22 (2), 138–144. 10.1002/(SICI)1098-2264(199806)22:2<138::AID-GCC8>3.0.CO;2-Y 9598801

[B36] Wainberg Z. A. Piha-Paul S. A. Luke J. Kim E. J. Thompson J. A. Britten C. D (2017). First-in-human phase 1 dose-escalation and expansion of a novel combination, anti-CSF-1 receptor (cabiralizumab) plus anti-PD-1 (nivolumab), in patients with advanced solid tumors. In 2017 Annual Meeting of the Society for Immunotherapy of Cancer. National Harbor, MD.

[B37] Yachida S. Iacobuzio-Donahue C. A. (2009). The pathology and genetics of metastatic pancreatic cancer. Arch. Pathol. Lab. Med. 133 (3), 413–422. 10.1043/1543-2165-133.3.413 19260747

[B38] Yamamoto H. Itoh F. Nakamura H. Fukushima H. Sasaki S. Perucho M. (2001). Genetic and clinical features of human pancreatic ductal adenocarcinomas with widespread microsatellite instability. Cancer Res. 61 (7), 3139–3144. 11306499

